# Dealing a Neonate with CHARGE Syndrome:Anaesthesia perspective of perioperative care

**DOI:** 10.12669/pjms.336.13558

**Published:** 2017

**Authors:** Khalid Maudood Siddiqui, Muhammad Ali Asghar, Amjad Nadeem

**Affiliations:** 1 Dr. Khalid Maudood Siddiqui, FCPS. Department of Anaesthesia, Aga Khan University Hospital Karachi, Pakistan; 2 Dr. Muhammad Ali Asghar, FCPS. Department of Anaesthesia, Aga Khan University Hospital Karachi, Pakistan; 3 Dr. Amjad Nadeem, FCPS. Department of Anaesthesia, Aga Khan University Hospital Karachi, Pakistan

**Keywords:** Neonate, CHARGE Syndrome, Choanal Atresia, Heart defects

## Abstract

CHARGE syndrome is a condition that can disturb numerous areas of human body. As an abbreviation CHARGE stands for: coloboma, heart defects, atresia choanae, and retardation of growth, genital, and ear abnormalities. The configuration of malformations differs among individuals with this disorder, and the various health issues can be life-threatening during infancy and childhood. Affected individuals typically have several main features or a combination of major and minor appearances. Here we are presenting a case report of a neonate with CHARGE syndrome who underwent successful repair of choanal atresia under general anaesthesia with invasive monitoring.

## INTRODUCTION

CHARGE syndrome is a genetic disorder with autosomal dominance involved in the mutated gene CHD7 on chromosome 8.^1^ The abbreviation “CHARGE” characterizes the association of coloboma, heart anomalies, choanal atresia, and retardation of growth, genital and ear anomalies.^1^ These often exist in various blends and to varying degrees.

## CASE PRESENTATION

A neonate weighing 2.2 kilogram term male was born to Gravida 1, mother via normal vaginal delivery in the remote area. After birth, the baby developed respiratory inefficiency with cyanosis and baby was referred to us. When baby was reported to emergency department his initial saturation was 78% on pulse oxymetry without oxygen supplementation. The baby was shifted to Neonatal Intensive Care Unit (NICU) for further care. During further checkup in the NICU, it was observed that baby has intermittent tet spells and cyanosis with rapid drop of saturation. Moreover, it was observed that baby was facing difficulty in breathing with nose, chest was retracted when the child was breathing through mouth or crying and furthermore there was slight evidence of coloboma of eyes was noticed. A 4 French nasogastric tube was tried to pass his nose bilaterally to rule out choanal atresia and it failed on that occasion.

The child’s airway was secured through a soft silicone made nipple. The end of the nipple was fully cut, and put in the child’s oral cavity to enable the oxygenation and ventilation. The resected nipple was protected with adhesive tape and thus an airway was formed. Supplemental oxygen, prophylactic antibiotics, and intravenous fluids were also on board.

In cardiovascular examination a systolic thrill anteriorly along the left sternal border was observed. A harsh systolic ejection murmur (SEM) over the pulmonic area and left sternal border was heard. His pre-cardial echo was done which showed tiny ventriculo septal defect (VSD) and slight pulmonary stenosis with right ventricle out flow tract obstruction.

Respiratory rate was observed 40 to 55 breaths/minutes, with no extra apparent signs of respiratory distress, the neonate needed 40% FiO2 to maintain up to 90% oxygen saturation. The recurrent cyanosis was resolved when the baby became naturally glowing on cry, and rest of all workup was insignificant. His chest X-ray was normal, other investigation like; septic markers, Complete blood count, C - reactive protein and blood culture were also sent and provisional diagnosis of CHARGE Syndrome was made.

**Fig.1:**
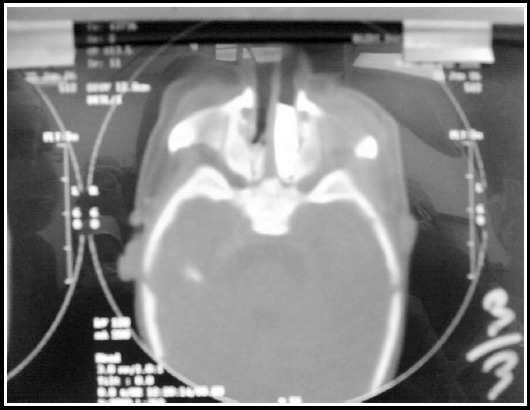
CT scan of the patient shown obstruction on both side of choanae.

On second day to confirm the diagnosis Fluoroscopy/ Choanography and CT Paranasal sinuses and nose were also done. Fluoroscopy exposed failure of contrast run from nose into nasopharynx, while CT Para nasal sinuses established the occurrence of bilateral bony Choanal Atresia (Fig.1). An ENT and cardiothoracic surgery consult was pursued for early surgical repair of the bilateral choanal atresia and possible Blalock–Taussig shunt surgery. But cardiac surgery was deferred till further growth of child.

Surgical correction of bilateral Choanal Atresia was planned and anaesthesia consult was given. Routine preoperative anaesthesia evaluation was done, and high risk consent was taken and necessary preparations were done with arrangement of blood product with cardiac surgery team on board.

Initial anaesthesia plan was to give inhalational induction with controlled mode of ventilation with both ultrasound guided arterial and central venous line insertion. After taking the patient in the operating room standard monitoring was applied with ECG, NIBP, Pulse oximetry and end tidal CO_2._ We provided anaesthesia with inhalational sevoflurane 2% with oxygen, we assessed his mask ventilation first, which was not difficult and after assurance of proper mask ventilation with confirmation of end tidal CO2 and rising of chest with ventilation, injection Atracurium 0.5mg/kg for muscle relaxation and injection Fentanyl 2µg/kg for analgesia were given in already placed 24G intravenous cannula on his left hand. After complete muscle relaxation, airway was secured with size three endotracheal tube and controlled mode of ventilation was achieved. We have placed ultra sound guided invasive lines in right femoral artery and vein for blood pressure and central venous pressure monitoring.

Surgical discovery was bilateral whole Choanal Atresia, which was partial bony and membranous on both sides and fixed with surgically by an endoscopic transnasal approach.

**Fig.2:**
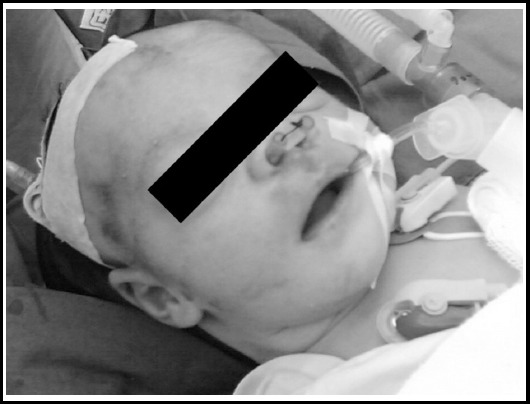
Bilateral surgical correction, showing stents in place.

After surgery 14 French size, Nelaton tube was inserted bilaterally and stitched anteriorly with sutures (Fig.2). Postoperatively patient remained stable and was discharged from hospital on 3^rd^ post-operative day. At the time of discharge, the baby was on mother feed and the Nelaton tube was removed after three weeks of surgery.

## DISCUSSION

B.D. Hall first described the CHARGE association in about 17 children who had choanal atresia.^2^ During the same year, H.M. Hittner described 10 children who had choanal atresia as well as coloboma, congenital heart defect, and hearing loss.^3^ In 1998, an expert group described the major classical 4C´s: Choanal atresia, Coloboma, characteristic ear and Cranial nerve anomalies and minor criteria of CHARGE syndrome. Individuals with all three or four major characteristics and three minor characteristics are likely to have CHARGE syndrome.^4^

Separately from choanal atresia cleft palate/lip are up to 56% of the victims, and associated with upper airway abnormalities as well.^5^ Up to 50% of the patients required tracheotomy not only for associated airway abnormalities but also for retention of saliva, chronic aspiration and swallowing disorders.^6^ Choanal atresia is a congenital unilateral or bilateral bony or membranous obstruction of the nasopharynx which can contribute to respiratory distress or asphyxia. Most of the time bilateral choanal atresia is present with respiratory crisis because newborn babies are required to breathe through nose. Certainly the reflex to assist breathing through the open mouth in response to nasal obstruction only developed weeks to months after birth, although an infant will breathe through mouth, if the mouth is opened either during crying or if an artificial oral airway is implanted.^7^ Therefore, neonates with bilateral choanal atresia used to exhibit a recurrent change in oxygenation, became cyanosed during quiet periods, and returned their normal colour when they cried. Choanal atresia may be found as an isolated abnormality but 60–70% of cases are associated with other congenital defects.^8^

There are higher chances to fail the permission of a soft transnasal catheter through nares in a suspected case and it is also considered as a diagnostic tool for the congenital choanal atresia. The reason to failure may be due to the hindrance of turbinate or adenoids in the passage, some authors support auscultation over the nostrils to assess the airflow.^7^ However, the current investigation of choice is a combination of nasal endoscopy and CT scanning. The CT scan indicates whether the atresia is membranous or bony and demonstrates the thickness in addition to excluding other differential diagnoses such as encephaloceles or dermoids. In our patient, the diagnosis is suspected with early presentation of respiratory obstruction soon after birth, failure to pass a transnasal catheter and later confirmed by endoscopic and CT scan findings.

Difficult or failed ventilation and endotracheal intubation are amongst the situations that anesthesiologists fear most. Patients with choanal atresia are considered to be at high risk for experiencing difficulty with anesthesia. Both complete and partial nasopharyngeal obstruction exacerbates airway obstruction with relaxation of upper airway tone after sedation or induction of general anesthesia. Inhaled anesthetics are preferred for the induction of anesthesia, particularly during the neonatal period, when the airway is difficult to intubate. Choanal atresia is also considered, and it is recommended that muscle relaxant agents be administered, if necessary, after the airway is secured in patients with no ventilation problem.^9^ Besides the airway problems, most children with CHARGE syndrome have cranial nerve abnormalities too. Pertinent problems are those with the glossopharyngeal and vagus nerves, which are responsible for innervating the pharynx and larynx and permit swallowing to occur.^10^

Preliminary preparation before administration in patients with choanal atresia who are anticipated to have a difficult airway makes the intubation procedure easier and reduces complications that might arise. Tubes of all types and diameters, a laryngoscope and various blades, Magill pliers, drugs, and equipment needed for cardiopulmonary resuscitation should be made available. Percutaneous and surgical tracheostomy kits and equipment, such as a fiber optic bronchoscope, video laryngoscope, LMA and I-gel, should also be made available, if possible, as preliminary preparation for difficult intubation. A previous case report defines successful airway management with a laryngeal mask airway in a patient with CHARGE syndrome and where the Cormack Lehane score was IV in direct laryngoscopy.^11^

Complications such as laryngospasm, bronchospasm, desaturation, and re-intubation were observed in the patients, but these were not unexpected complications. They can develop due to the manoeuvers, the instruments used during difficult intubations. It is important to be prepared for any complications that may arise.

Anesthesiologists must be concerned about anticipated post operated airway difficulties which are common in children with CHARGE syndrome. In a study 35% of anesthetic procedures resulted in post-operative airway challenges. The most common were decreased oxygen saturation, obstruction from excessive secretions with desaturations requiring management, prolonged wheezing, heart arrhythmias, decreased respiratory rate, stridor and failed extubation. Post-operative airway difficulties were most likely to occur surgical procedures involving the cardiovascular and/or the gastrointestinal systems, or after diagnostic scopes.^12^ Assumed these risks, children with CHARGE syndrome should be monitored longer after surgery than the general paediatric population.

The patients with CHARGE syndrome have higher chances to need prolonged post-operative mechanical ventilation. It is recommended to do mechanical ventilation in higher supervision. Ambulatory anaesthesia is not recommended due to the significantly higher risk for post-anaesthetic adverse airway events.

## CONCLUSION

Individuals with CHARGE syndrome need intensive care, suitable to their particular features. Furthermore, early intervention and appropriate medical and surgical management specifically anesthesia care needs are imperious. Although there are many problems associated to children with CHARGE Syndrome, and anesthesia management is challenging and require expert skills. Early recognition and intervention can definitely improve the survival and outcome benefits.

### Authors’ Contribution

**KMS:** Conceived, concept and writing of manuscript.

**MAA:** Did data collection and manuscript writing.

**AN:** Did the literature search.
